# miR-133a-5p Inhibits Glioma Cell Proliferation by Regulating IGFBP3

**DOI:** 10.1155/2022/8697676

**Published:** 2022-08-02

**Authors:** Xinzhi Yang, Dong Chen, Jiliang Hu, Qingsuo Zhao, Xing Fu, Wen Lv

**Affiliations:** ^1^Department of Neurosurgery, Shenzhen Hospital of Southern Medical University, Shenzhen, Guangdong, China; ^2^Department of Neurosurgery, Shenzhen Yantian District s People s Hospital, Shenzhen, Guangdong, China; ^3^Department of Neurosurgery, Shenzhen People's Hospital, Shenzhen, Guangdong, China; ^4^Guangdong Engineering Technological Research Center for Nervous Anatomy and Related Clinical Applications, Zhongshan, China; ^5^Department of Neurosurgery, Changsha Central Hospital of University of South China, Changsha, Hunan, China; ^6^Department of Neurosurgery, The First Affiliated Hospital, Southern University of Science and Technology, Shenzhen, Guangdong, China

## Abstract

**Objective:**

This research aims to investigate the expression of miR-133a-5p in glioma tissues and its impact on glioma cell proliferation.

**Methods:**

Fluorescence-quantitative PCR was used to detect the expression of miR-133a-5p in 25 cases of glioma and adjuncent tissues. CCK-8 and colony formation analyses were used to evaluate the impact of transfection with miR-133a-5p inhibitors or mimics on glioma cell growth and colony formation. The IGFBP3 (insulin-like growth factor-binding protein-3) and miR-133a-5p binding sites were predicted using Starbase, and the miR-133a-5p binding capacity with 3'UTR of IGFBP3 gene was determined using a luciferase gene reporter system. Following transfection with miR-133a-5p mimics or inhibitors, the IGFBP3 protein expression in glioma cells was determined by western blotting. The colony formation assay was applied to evaluate the influence of IGFBP3 overexpression on the miR-133a-5p in glioma cell proliferation. For assessment of the IGFBP3 expression in glioma tissues and prognosis, TCGA database was employed.

**Results:**

The expression of miR-133a-5p was considerably reduced in glioma tissue compared to adjuncent control tissue. In addition, miR-133a-5p expression decreased with increasing glioma malignancy. Glioma cell growth and colony formation were reduced after miR-133a-5p mimics were transfected, while transfection of miR-133a-5p inhibitors had a reverse impact. The expression of IGFBP3 was affected by miR-133a-5p by binding to its 3'UTR region. Additional study demonstrated that the overall survival (OS) of subjects with increased IGFBP3 expression was considerably lower compared to patients with decreased IGFBP3 expression. The IGFBP3 overexpression effectively counteracts the glioma cell proliferation-inhibiting impact of miR-133a-5p.

**Conclusion:**

miR-133a-5p acts as a glioma tumor suppressor gene. It reduces glioma cell proliferation by modulating IGFBP3 and could be a target for glioma therapy.

## 1. Introduction

Glioma arises from glial cells' surrounding neurons and is the most prevalent tumor of the central nervous system (CNS) found in clinical settings. Among all gliomas, glioblastoma has an extremely high recurrence rate and is responsible for roughly 80% of all aggressive brain tumors. Glioblastoma is one of the malignant tumors with the poorest prognostic outcome [1, 2]. Despite significant advances in surgery, radiotherapy, and chemotherapy in recent decades, the prognosis of glioma remains poor, with a mean survival time of only 14.6 month [3, 4]. Various fundamental and clinical research studies have revealed that glioma is a polygenic illness and that its onset and progression are controlled by numerous genes [5, 6]. Therefore, understanding the interaction among relevant factors at the level of gene regulation and looking for additional molecular candidate genes have become important for glioma therapy.

MicroRNA (miRNA) is a kind of non-coding single-stranded RNA (ncRNA) that can attach to the 3′UTR domain of target genes and restrict gene expression at the post-transcriptional level, ultimately leading to mRNA breakdown and translational reduction [7, 8]. Prior research has shown that miRNA plays a significant role in controlling cell differentiation, proliferation, apoptosis, and tumor growth and development [9]. miRNAs are implicated in all tumor-related activities, including immunological response and angiogenesis. They can enhance or suppress cancer growth by blocking the production of specific molecules in signaling networks [7, 10]. For instance, miRNA-451 can modulate the NF-B signaling cascade by activating IKK*β*, reducing glioma cell proliferation both *in vivo* and *in vitro* [11]. miR-23b-5p increases sensitivity of glioma to temozolomide treatment by negatively regulating TLR4 expression [12]. These findings suggest that miRNAs play important roles in the incidence, drug resistance, and progression of glioma.

Recent research has indicated that miR-133a-5p is a tumor suppressor gene that is poorly expressed in a range of tumor tissues and plays an antitumor role via modulating downstream target genes in gastric cancer, bladder cancer, and non-small-cell lung cancer. For example, miR-133a-5p is modestly expressed in gastric cancer cell lines and tissues. The analysis of its molecular mechanism has revealed that miR-133a-5p suppresses metastasis and cell growth while promoting apoptosis by targeting TCF4 [13]. Furthermore, in prostate cancer cells, miR-133a-5p reduces cellular invasiveness and proliferation via targeting the androgen receptor (AR) [14]. Other molecules, such as circRNA circP4HB increases the metastasis and invasiveness of non-small-cell lung cancer (NSCLC) by sponging miR-133a-5p, can also control the regulating effect of miR-133a-5p to exert biological functions in tumor cells [15].

However, the expression and regulatory effects of miR-133a-5p in glioma tissue is unknown. This study sought to determine the expression of miR-133a-5p in glioma cells and tissues. Moreover, the impact of miR-133a-5p overexpression or inhibition on glioma cell proliferation was investigated. The regulatory link between IGFBP and miR-133a-5p was validated. This study will provide a theoretical foundation for clinical treatment of glioma.

## 2. Materials and Methods

### 2.1. Research Materials

Twenty-five participants that underwent glioma excision at our hospital between June 2020 and January 2021 were included in this study. The glioma and para-carcinoma specimens (∼5 cm away from tumor tissue edge) were obtained and preserved in the freezer at −80°C. There was no preoperative chemotherapy, radiotherapy, immunotherapy, targeted therapy, or other relevant treatments. All participants signed an informed consent form. All specimens were obtained and operated following the ethical standards of clinical trials.

### 2.2. Cell Culture and Transfection

The Shanghai Cell Bank (Chinese Academy of Sciences) provided normal astrocyte (NHAS) cells and human glioma cell lines T98MG, U251, and U87. These cells were maintained in a DMEM containing 10% FBS. The culture conditions were 5% of CO_2_ concentration, incubation temperature of 37°C, relative humidity of 95%, and incubation in darkness. They were subcultured when the cells grew to an appropriate cell density (∼80% confluence). The cells at logarithmic growth were implanted into 6-well cell plates (1 × 10^6^ cells per well). The transfection was performed when the cell fusion reached about 60% as per the instruction of the Lipofectamine 2000 transfection kit.

### 2.3. Fluorescence-Quantitative PCR Detection

The TRIzol method was utilized for RNA extraction from para-carcinoma and glioma tissues. The glioma cell lines and concentration and purity of the RNA were determined using a spectrophotometer. Through a reverse transcription kit, the mRNA was reversedly-transcribed into cDNA. miR-133a-5p and internal reference U6 primers were added after the reverse transcription and then amplified in ABI fluorescence-quantitative PCR instrument. Each sample was repeated three times to quantify the relative expression level using the 2^−ΔΔC^T method.

### 2.4. CCK-8 for Cell Viability Detection

After transfection, the CCK-8 kit was utilized to detect the proliferative ability of glioma cells. Glioma cells in the log growth phase were paused and enumerated following transfection. Cells were kept in a 5% CO_2_ incubator and inoculated into a 96-well plate at 37°C with 100 *μ*L (1 × 10^3^ cells) per well. At four periods of time (24, 48, 72, and 96 h) following transfection, 10 *μ*l CCK-8 solution was poured into each well 2 h before detection, and a microplate reader was used to quantify the absorbance rate. Each sample was repeated in triplicate.

### 2.5. Colony Formation Assay

After transfection, 0.25% trypsin was applied to digest the glioma cells in the log growth phase, dispersed into a single-cell suspension, and centrifuged at 1000 rpm at room temperature for 5 min. The supernatant was discarded, and the remaining cells were enumerated after resuspension. A total of 500 cells were plated into a six-well plate, the media were replaced every two to three days, and cell development was constantly monitored. After around 2 weeks, the medium was removed, 4% paraformaldehyde was introduced for 15 min, and the cell staining was performed using 1% crystal violet solution. Cell pictures were collected. The cell mass with ≥50 cells was taken as the number of colonies, and the colony formation rate was analyzed. The procedure was repeated three times.

### 2.6. Dual-Luciferase Reporter Gene

Starbase online prediction software was applied to identify the probable downstream miR-133a-5p target genes. It was found that miR-133a-5p had a binding affinity with the 3′-UTR region of IGFBP, resulting in a dual-luciferase reporter gene vector formation. Glioma cells were harvested and plated at a density of 5 × 10^4^ cells per well in a 24-well plate. The no-load vector and reporter vector were prepared with miR-133a-5p mimics and control, respectively, to form a transfection mixture. After adding the transfection mixture to the cells, they were kept in a 37°C incubator with 5% CO_2_. The cells were cultured and kept in a complete medium for 48 h after being cultured for 12 h. The cells were lysed per the Promega dual-luciferase reporter gene detection kit protocol, and luciferase activity was determined by adding a fluorescent substrate.

### 2.7. Western Blot Analysis

The expression of IGFBP protein in glioma cells transfected with miR-133a-5p inhibitors or mimics was analyzed using a western blot technique. Equal amounts of cells from each transfection group were taken, and the SDS lysis method was applied to extract the total protein. After protein denaturation, SDS-PACE electrophoresis was performed, and the membrane was transferred, and then it was blocked overnight with 3% BSA. The primary antibody (1 : 800 dilution) was applied and kept at 4°C overnight before washing the membrane. The HRP-labelled secondary antibody was used and incubated at room temperature for 1 h before washing the membrane and performing ECL chromogenic exposure. For the film after imaging, the absorbance values of each band were quantified using a density scanner.

### 2.8. Statistical Data Analysis

The SPSS software (v.19.0) was used for data analysis. The statistical data were described as mean ± SD. For independent samples, a *t-*test was used to look at measured values. The ANOVA was applied to search for differences between each group. Statistical significance was defined as a *P* value of less than 0.05.

## 3. Results

### 3.1. miR-133a-5p Was Expressed at Low Level in Glioma Tissues and Cell Lines

**Figure 1:**
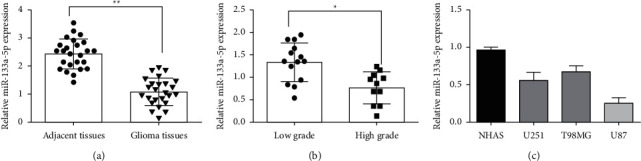
miR-133a-5p is expressed at low level in glioma tissues and cell lines. (a) The quantitative-fluorescence PCR assay identified the expression levels of miR-133a-5p in glioma and para-carcinoma tissues. (b) miR-133a-5p expression in the high and low-grade glioma tissues. (c) The expression levels of miR-133a-5p in glioma cell lines (^*∗*^*P* < 0.05 and ^*∗∗*^*P* < 0.01).

First, fluorescence-quantitative PCR determined the expression of miR-133a-5p in glioma and para-carcinoma tissues. The analysis indicated that glioma tissue had less miR-133a-5p than para-carcinoma tissue (Figure 1(a)). The glioma tissues were then subdivided into high-grade and low-grade glioma tissues. The findings demonstrated that the miR-133a-5p expression reduced as the malignant degree of the glioma increased (Figure 1(b)). Furthermore, compared to normal astrocyte (NHAS) cells, the expression level of miR-133a-5p was lowered in glioma cell lines, particularly in U87 cells (Figure 1(c)). These results suggested that the expression of miR-133a-5p reduced in glioma cells and tissues.

### 3.2. The Effect of miR-133a-5p on Glioma Cell Proliferation

**Figure 2:**
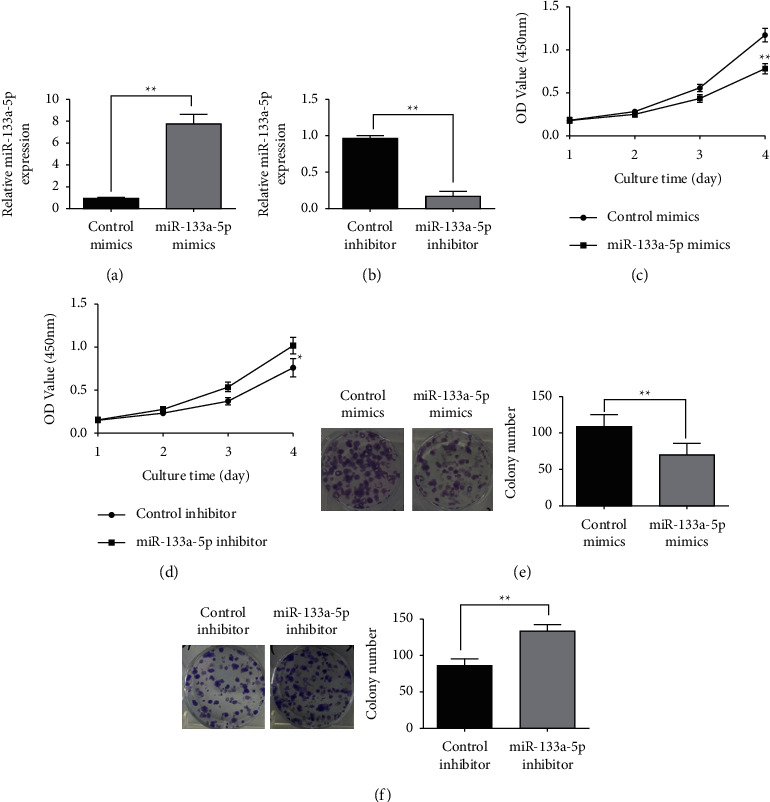
Effect of miR-133a-5p on glioma cell proliferative ability. (a, b) Fluorescence-quantitative PCR detected the miR-133a-5p expression level in U87 and T98MG cells following transfection with miR-133a-5p mimics or inhibitors. (c, d) In U87 and T98MG cells, the MTT assay was used to examine the transfection of miR-133a-5p mimics or inhibitors that affected cellular proliferation. (e, f) A colony formation test was utilized to examine the impact of transfection of miR-133a-5p mimics or inhibitors on colony formation ability in U87 and T98MG cells (^*∗*^*P* < 0.05 and ^*∗∗*^*P* < 0.01).

The initial experimental results suggested that the expression of miR-133a-5p was lower in U87 cells than in T98MG cells. U87 cells were transfected with miR-133a-5p and control mimics, and fluorescence-quantitative PCR detection revealed that miR-133a-5p mimic transfection substantially enhanced the miR-133a-5p expression level in cells as compared to the control (Figure 2(a)). In addition, T98MG cells were transfected with miR-133a-5p inhibitor and the control. Compared to the control, transfection of miR-133a-5p inhibitor significantly reduced miR-133a-5p expression in the cells (Figure 2(b)). Compared to the control, the miR-133a-5p mimic transfection decreased cell growth considerably in U87 cells, as revealed by MTT assay findings (Figure 2(c)). The control mimics, transfected with miR-133a-5p inhibitor, greatly increased cell proliferation in T98MG cells (Figure 2(d)). Furthermore, the colony formation experiment indicated that miR-133a-5p mimic transfection considerably lowered the competence of U87 cells to form colonies when compared to the control (Figure 2(e)). Transfection with miR-133a-5p inhibitor substantially elevated the ability of T98MG cells to form colonies when compared to the control (Figure 2(f)).

### 3.3. miR-133a-5p Regulates the IGFBP3 (Insulin-Like Growth Factor-Binding Protein-3) Expression in Glioma Cells

**Figure 3:**
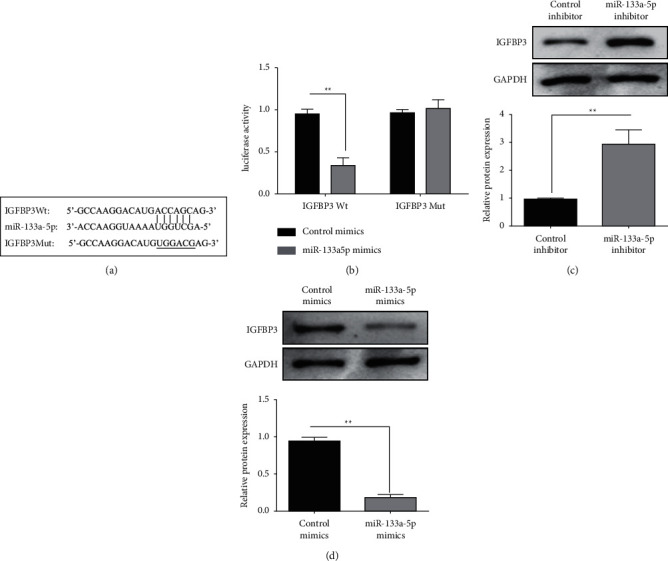
The IGFBP3 expression in glioma cells is regulated by miR-133a-5p. (a) miR-133a-5p had a binding site with IGFBP3. (b) The dual-luciferase reporting assay measured miR-133a-5p and IGFBP3 binding activity. (c) The western blot measured the transfection of miR-133a-5p mimics or inhibitors on IGFBP3 expression in U87 and T98MG cells (^*∗∗*^*P* < 0.01).

The TargetScan prediction software study revealed that miR-133a-5p had a binding site with IGFBP3 (Figure 3(a)). The luciferase activity of U87 cells in the miR-133a-5p mimic and IGFBP3 Wt co-transfection group was substantially lower than that in the control and IGFBP3 Wt co-transfection group, according to the findings of the dual-luciferase report analysis. Compared with the control mimics and IGFBP3 Mut co-transfection group, the luciferase activity of U87 cells in the miR-133a-5p mimic and IGFBP3 Mut co-transfection group did not change significantly (Figure 3(b)). According to western blotting assay results, IGFBP3 protein expression in U87 cells was decreased in the miR-199a-5p mimic group compared to the control group (Figure 3(c)). In comparison to the control group, the IGFBP3 protein expression in T98MG cells was dramatically increased in the miR-199a-5p inhibitor group (Figure 3(d)).

### 3.4. IGFBP3 Was Expressed in Gliomas and Could Be a Biomarker for Patient Prognosis

**Figure 4:**
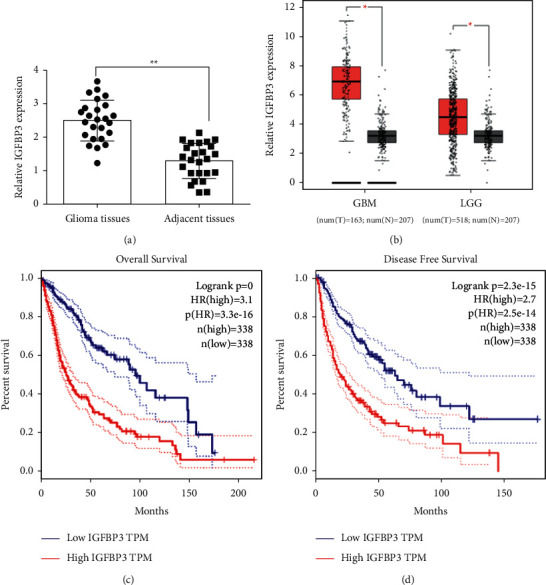
IGFBP3 was overexpressed in gliomas and could be exploited as a prognostic biomarker. (a) IGFBP3 expression levels in gliomas, and para-cancerous tissues determined by quantitative-fluorescence PCR. (b) The TCGA database was used to compare IGFBP3 expression level between GBM and LGG. (c, d) TCGA database was used to examine the association between IGFBP3 expression level, disease-free survival (DFS), and overall survival (OS) (^*∗∗*^*P* < 0.01).

To evaluate the expression of IGFBP3 in glioma, the expression of IGFBP3 in glioma and para-cancerous brain tissues was determined by fluorescence-quantitative PCR. The findings confirmed that IGFBP3 was overexpressed in glioma tissues compared to para-cancerous tissues (Figure 4(a)). Analysis of published studies from TCGA database on IGFBP3 expression in glioma revealed that IGFBP3 was strongly elevated in glioma tissues (low-grade glioma (LGG) and glioblastoma multiforme (GBM)) compared to para-cancerous tissues (Figure 4(b)). Significantly shorter overall survival (OS) and disease-free survival (DFS) were seen in patients with high IGFBP3 expression compared to those with low IGFBP3 expression (Figures 4(c) and 4(d)).

### 3.5. IGFBP3 Overexpression Can Mitigate the miR-133a-5p Inhibitory Effect on the Proliferation of Glioma Cells

**Figure 5:**
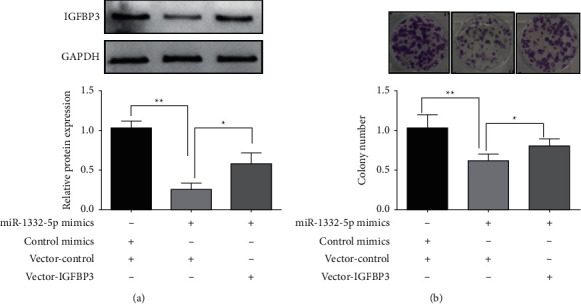
IGFBP3 overexpression mitigates the inhibitory effect of miR-133a-5p on the proliferation of glioma cells. (a) Effect of overexpression of IGFBP3 on upregulation of IGFBP3 protein expression in miR-199a-5p cells. (b) IGFBP3 overexpression mitigates the inhibitory effect of miR-133a-5p on glioma cell proliferation (^*∗∗*^*P* < 0.01 and ^*∗*^*P* < 0.01).

The western blot analysis showed that the miR-133a-5p mimics and the vector group could reduce IGFBP3 expression, confirming IGFBP3 gene suppression by miR-133a-5p. Following transfection with an overexpression vector (Vector-IGFBP3) that overexpressed IGFBP3, the inhibition of IGFBP3 by overexpression of miR-133a-5p was dramatically reduced in the miR-133a-5p mimic group (Figure 5(a)). Compared to the vector group and miR-133a-5p mimics, both groups effectively suppressed cell colony formation. Similarly, the inhibitory effect of miR-133a-5p overexpression on cell colony formation was inhibited after transfection with an overexpression vector (Vector-IGFBP3) that overexpressed IGFBP3 (Figure 5(b)).

## 4. Discussion

Glioma is the most frequent recurrent malignant brain tumor. Due to the unsatisfactory effect of surgery, radiotherapy, and chemotherapy and its poor prognosis, gliomas seriously endanger human health [16, 17]. According to previous research, miRNAs contribute significantly to the transduction of intracellular signaling pathways, initiation, and progression of gliomas by modulating the expression of target genes and are a special class of prospective indicators in targeted therapies [18, 19]. miR-133a-5p was expressed at low level in glioma tissues, and its level of expression decreased substantially as glioma malignancy progressed, according to this study's results. Cell function experiments demonstrated that miR-133a-5p overexpression greatly decreased the glioma cell proliferation and colony formation, whereas the inhibition of miR-133a-5p had the opposite effect. Molecular mechanistic studies have reported the binding capacity of miR-133a-5p with the 3′-UTR region of IGFBP3 gene, influencing its expression. IGFBP3 overexpression can drastically counteract the inhibitory activity of miR-133a-5p on glioma cell growth. Therefore, miR-133a-5p could be utilized as a potential treatment target for glioma therapy.

The latest evidence has suggested that abnormal miRNA expression is closely related to the development and proliferation of tumor cells. For example, the overexpression of miR-191 can enhance cell proliferation both *in vivo* and *in vitro* by negatively regulating the expression of NDST1 in human glioblastoma tissues and cells [20]. mir-758-5p targets ZBTB20 expression, which reduces glioblastoma invasion, migration, and proliferation [21]. miR-133a-5p was found to be weakly expressed in glioma tissues. The cell proliferation was inhibited considerably by miR-133a-5p overexpression. miR-133a-5p suppression enhanced cell proliferation. The target gene of miR-133a-5p was identified as IGFBP3 using bioinformatics predicted analyses in this study, which aimed to expand our understanding of the mechanism of miR-133a-5p in glioma cell proliferation.

IGFBP3 belongs to a class of intracellular molecules with numerous regulatory functions that play various roles in many cancers. IGFBP3 is abundantly expressed in nasopharyngeal cancer tissues, which correlates with poor prognosis and tumor metastasis. Overexpression of IGFBP3 can promote cell proliferation and migration [22]. In cervical cancer, IGFBP3 inhibits tumor angiogenesis by intracellular regulation of THBS1 expression [23]. In hepatocellular carcinoma cells, overexpression of IGFBP3 induces apoptosis of hepatocellular carcinoma cells and reduces colony formation [24]. This study demonstrated that IGFBP3 is the target gene of miR-133a-5p and that increasing miR-133a-5p can inhibit glioma cells from expressing IGFBP3 protein. In this study, an IGFBP3 overexpression vector and a miR-133a-5p mimic were transfected in glioma cells. It was revealed that IGFBP3 overexpression reverses the inhibitory effect of miR-133a-5p on glioma cell proliferation. This work established the role of IGFBP3/miR-133a-5p axis in the glioma cell proliferation at the cellular level *in vitro*. More *in vivo* research is required for further exploration.

In conclusion, the proliferative potential of glioma cells is inhibited by miR-133a-5p, which reduces IGFBP3 synthesis. The results of this study encourage the development of novel clinical therapies by targeting miR-133a-5p.

## Data Availability

The data used to support the findings of this study are available from the corresponding author upon request.
